# Supplementary material for: Tailoring COVID-19 Vaccination Strategies in High-Seroprevalence Settings: Insights from Ethiopia
Source: Vaccines (Basel). 2024 Jul 5;12(7):745. doi: 10.3390/vaccines12070745 (PMC11281643; doi:10.3390/vaccines12070745)
Supplement: Supplementary file 1 [file vaccines-12-00745-s001.zip › vaccines-3083745-supplementary.pdf]

Tailoring COVID-19 Vaccination Strategies in High-Seroprevalence Settings: Insights from Ethiopia

Esayas Kebede Gudina, Kira Elsbernd, Daniel Yilma, Rebecca Kisch, Karina Wallrafen-Sam, Gemeda Abebe, Zeleke Mekonnen, Melkamu Berhane, Mulusew Gerbaba, Sultan Suleman, Yoseph Mamo, Raquel Rubio-Acero, Solomon Ali, Ahmed Zeynudin, Simon Merkt, Jan Hasenauer, Temesgen Kabeta Chala, Andreas Wieser and Arne Kroidl

Supplementary materials

I. Baseline characteristics of vaccinated health professionals

A total of 310 healthcare workers (HCWs) who received Covishield vaccine during the first round of COVID-19 vaccination campaign at Jimma University (07 to 12 April 2021) were recruited. Of these, 259 attended the second round; five of them did not receive the vaccine and were excluded as a result (Table S1).

II. Vaccine adverse events and post-vaccine antibody response

Post vaccine antibody assay was conducted at a median of 91 days (IQR: 86 – 97) after the first dose of the vaccine with range of 73 to 142 days; nearly 75% were tested within 80 to100 days after vaccination.

Overall, 67.3% of the participants reported vaccine adverse events after the first dose of the vaccine. Fatigue (42.9%), headache (37.0%), and injection site pain (34.7%) were the most commonly reported adverse effects. Most of the adverse effects (94.3%) occurred in the first two days. Only 9.4% of the adverse effects were self-reported as severe but none of them needed hospitalization. There is no difference in the incidence of the adverse events by sex; however, the adverse events were reported more by the younger participants (mean age for those with symptoms 31.5 years versus 34.1 years for those not reporting symptoms, P=0.017).

Twenty-seven participants developed COVID-19 related symptoms after the last visit. Sore throat, nasal congestion, and fatigue were the most commonly reported symptoms.

However, only two of them were tested with RT-PCR for SARS-CoV-2, one of which was positive (Table S2).

Table S1. Baseline (pre-COVID-19 vaccination) characteristics of study participants.

Sex, N (%)	
Female	80 (31.5)
Male	174 (68.5)
Age in years, Mean (SD)	32.3 (7.9)
Profession, N (%)	
Nurse	79 (31.1)
Medical doctors (all categories)	72 (28.3)
Teaching staff	37 (14.6)
Laboratory professional	12 (4.7)
Pharmacist	11 (4.3)
Other health professionals	17 (6.7)
Others	26 (10.2)
Ever been tested with PCR for COVID-19, n (%)	99 (39)
Positive PCR	63 (63.6)
Negative PCR	36 (36.4)
Recent place of work, N (%)	
Inpatient department	65 (25.6)
Operation room	41 (16.1)
Outpatient department	38 (15.0)
Emergency room	24 (9.4)
Delivery room	15 (5.9)
Chronic follow-up unit	11 (4.3)
Intensive care unit	8 (3.1)
COVID-19 treatment center	8 (3.1)
Other	44 (17.3)
Preexisting medical condition, n (%)	16 (6.3)
SARS-CoV-2 anti-nucleocapsid antibody, N (%)	158 (62.2)
SARS-CoV-2 anti-spike antibody, N (%)	165 (65)

PCR – polymerase chain reaction

Table S2. Post-COVID-19 vaccine self-reported adverse events and SARS-CoV-2 serostatus.

Days since COVID-19 vaccination, median (IQR)	91 (11)
Self-reported adverse event after first dose, N (%)	
Yes	171 (67.3)
No	83 (32.7)
Types of adverse reaction, N (%)	
Fatigue	109 (42.9)
Headache	94 (37)
Injection site pain	88 (34.7)
Muscle pain	77 (30.3)
Fever	51 (20.1)
Other *	23 (9.1)
Day of ADR onset (n=171), N (%)	
Day 1	129 (75.4)
Day 2	34 (19.9)
Day 3 and after	8 (4.7)
Self-reported severity of the ADR (n=171), N (%)	
Mild	103 (60.2)
Moderate	52 (30.4)
Severe	16 (9.4)
COVID-19 related symptoms since vaccination, N (%)	27 (10.6%)
Symptom type	
Sore throat	10
Nasal congestion	10
Fatigue	7
Muscle pain	5
Frequent sneezing	5
Post-vaccine anti-NC antibody, N (%)	
Positive	169 (66.5)
Negative	85 (33.5)
Post-vaccine anti-Spike protein antibody, N (%)	
Positive	248 (97.6)
Negative	1 (0.4) †
Not tested**	5 (2)

* Nausea/vomiting, abdominal pain, diarrhea, sleep disorder, joint pain

** Specimen amount not sufficient

† 36 years old man who was negative for both anti-NC and anti-Spike protein both at baseline and post-vaccination

III. Vaccine break through infection and seroreversion after vaccination

Among 96 participants who were negative for anti-nucleocapsids at baseline, 22 (22.9%) became positive post-vaccine (probably due to *vaccine breakthrough infection or*

seroconversion after window period during first round). However, only two of them reported COVID-19 like symptoms since they received vaccination. On the other hand, 11 of the 158 participants (7%) who had anti-nucleocapsid antibody at baseline became negative for it after three months (*sero-reversion*).

IV. Cost-Effectiveness Analysis of SARSCoV- 2 Vaccination Strategies in Ethiopia

Definition of Vaccination Strategies

We aim to assess the cost per percentage point increase in the percentage of the adult population of Ethiopia with anti-spike levels above 275 BAU/mL relative to a no-vaccination scenario for three vaccination strategies:

1. **The 2-Dose Strategy**: Giving two vaccine doses per person until vaccines run out.
2. **The 1-Dose Strategy**: Giving one dose per person until vaccines run out.
3. **The Individual Testing Strategy**: While vaccine supplies last, performing an antibody test on each vaccine-eligible individual and administering one dose if their test indicates a previous infection and two doses if not.

The 275 BAU/mL cutoff was chosen based on Merkt et al. [1]. It is also reasonably close to 264 BAU/mL, which is the value identified by Feng et al. [2] as corresponding to 80% vaccine efficacy against symptomatic disease. Hereafter, for convenience, we refer to having anti-spike levels above 275 BAU/mL as "being immune".

Throughout the following analysis, we make the following simplifying assumptions: individual antibody tests have perfect specificity; the antibody response to one infection and one dose of a given vaccine is equivalent to the antibody response to two doses of that same vaccine; and no one in the population of interest has been infected more than once.

Parameter Definitions

Table S3: Cost-utility analysis parameters and their sources.

Variable	Description	Value	Source
N_{vax}	Number of available vaccine doses	$2.0 \cdot 10^6$	[3]
N_{pop}	Number of vaccine-eligible persons	$72.2 \cdot 10^6$	[4]
p	Proportion of vaccine-eligible persons who have been infected	0.613	Calculated directly from study data*
i	Percent of unvaccinated, previously infected persons who are immune	14.2%	Calculated directly from study data
v	Percent of once-vaccinated, not previously infected persons who are immune	AstraZeneca: 11.6%<br>Pfizer: 20.3%	For AstraZeneca, study data; for Pfizer, [5]**
d	Percent of those with two past exposures (infection and vaccination or two vaccinations) who are immune	AstraZeneca: 95.5%<br>Pfizer: 100%	For AstraZeneca, study data; for Pfizer, [5] **
c_{vax}	Per-dose vaccine cost	AstraZeneca: $3<br>Pfizer: $17	[6]
c_{test}	Cost of performing one individual antibody test	$0 to $12	Free parameter
Se	Test sensitivity	80%, 95%, or 100%	[7] ***

* We set p to the proportion of study participants who tested positive for both anti-spike and anti-nucleocapsid antibodies prior to vaccination.

** Wheeler et al. [5] reported median and maximum anti-spike levels after each dose of the Pfizer vaccine in persons without previous infection. We assumed that the post-Pfizer antibody responses X were log-normally distributed with all data falling within two standard deviations of the mean to calculate $P(X > \log(275))$ based on these reported values.

*** According to diagnostics.roche.com [7], the sensitivity of the Elecsys Anti-SARS-CoV-2 S assay can range from 85.5% to 100% depending on test timing, so 80%, 95%, and 100% were chosen to represent the range of plausible sensitivity values.

Cost and Utility Equations

In the no-vaccination reference scenario, the percentage of the population of interest who are immune is the product of the previous infection prevalence p and the percent immune among unvaccinated, previously infected persons i, pi. For each of the three intervention scenarios, we calculate the percentage point increase in the population percentage immune, the "utility", by subtracting this reference level of immunity, pi, from the expected postintervention level of immunity.

Thus, for the 2-Dose Strategy, the utility U_{2D} and the total cost C_{2D}, respectively, are:

$$U_{\text{2D}} = \frac{N_{\text{vax}}}{2N_{\text{pop}}} d + \left(1 - \frac{N_{\text{vax}}}{2N_{\text{pop}}}\right) pi - pi = \frac{N_{\text{vax}}}{2N_{\text{pop}}} (d - pi)$$

$$C_{\text{2D}} = N_{\text{vax}} * c_{\text{vax}}.$$

The utility U_{1D} and total cost C_{1D} for the 1-Dose Strategy are:

$$U_{\text{1D}} = \frac{N_{\text{vax}}}{N_{\text{pop}}} (pd + (1-p)v) + \left(1 - \frac{N_{\text{vax}}}{N_{\text{pop}}}\right) pi - pi = \frac{N_{\text{vax}}}{N_{\text{pop}}} (p(d - v - i) + v)$$

$$C_{\text{1D}} = N_{\text{vax}} * c_{\text{vax}}.$$

And the utility U_{IT} and total cost C_{IT} for the Individual Testing Strategy are:

$$U_{\text{IT}} = \frac{N_{\text{vax}}}{N_{\text{pop}}(2 - p\text{Se})} d + \left(1 - \frac{N_{\text{vax}}}{N_{\text{pop}}(2 - p\text{Se})}\right) pi - pi = \frac{N_{\text{vax}}}{N_{\text{pop}}(2 - p\text{Se})} (d - pi)$$

$$C_{\text{IT}} = N_{\text{vax}} * c_{\text{vax}} + \frac{N_{\text{vax}}}{2 - p\text{Se}} * c_{\text{test}}.$$

We aim to minimize the cost per unit increase in utility. As such, we can find the threshold testing cost c^*_{test} below which the Individual Testing Strategy is preferable to the 2-Dose Strategy by finding the value of c_{test} for which $\frac{C_{\text{IT}}}{U_{IT}} = \frac{C_{\text{2D}}}{U_{\text{2D}}}$:

$$c^*_{\text{test}} = (2 - p\text{Se}) \left(c_{\text{vax}} \frac{U_{\text{IT}}}{U_{\text{2D}}} - c_{\text{vax}}\right) = c_{\text{vax}} p\text{Se}.$$

Thus, c^*_{test} is directly proportional to the prevalence of previous infection p, the test sensitivity Se, and the vaccine cost c_{vax}. It is independent of all other parameters (including the vaccine effectiveness parameters d and v).

Similarly, we can find the threshold testing cost $c^\dagger_{\text{test}}$ below which the Individual Testing Strategy is preferable to the 1-Dose Strategy by finding the value of c_{test} for which $\frac{C_{\text{IT}}}{U_{\text{IT}}} = \frac{C_{\text{1D}}}{U_{\text{1D}}}$:

$$c^\dagger_{\text{test}} = (2 - p\text{Se}) \left(c_{\text{vax}} \frac{U_{\text{IT}}}{U_{\text{1D}}} - c_{\text{vax}}\right) = \frac{c_{\text{vax}}(d - pi)}{p(d - v - i) + v} - c_{\text{vax}}(2 - p\text{Se}).$$

As seen in the above equations, all key results are independent of the parameters N_{vax} and N_{pop}.

Additional Results

The results for the case in which Se = 1 are in the main manuscript. Below are the results for Se = 0.95 (Figure S1) and Se = 0.80 (Figure S2).

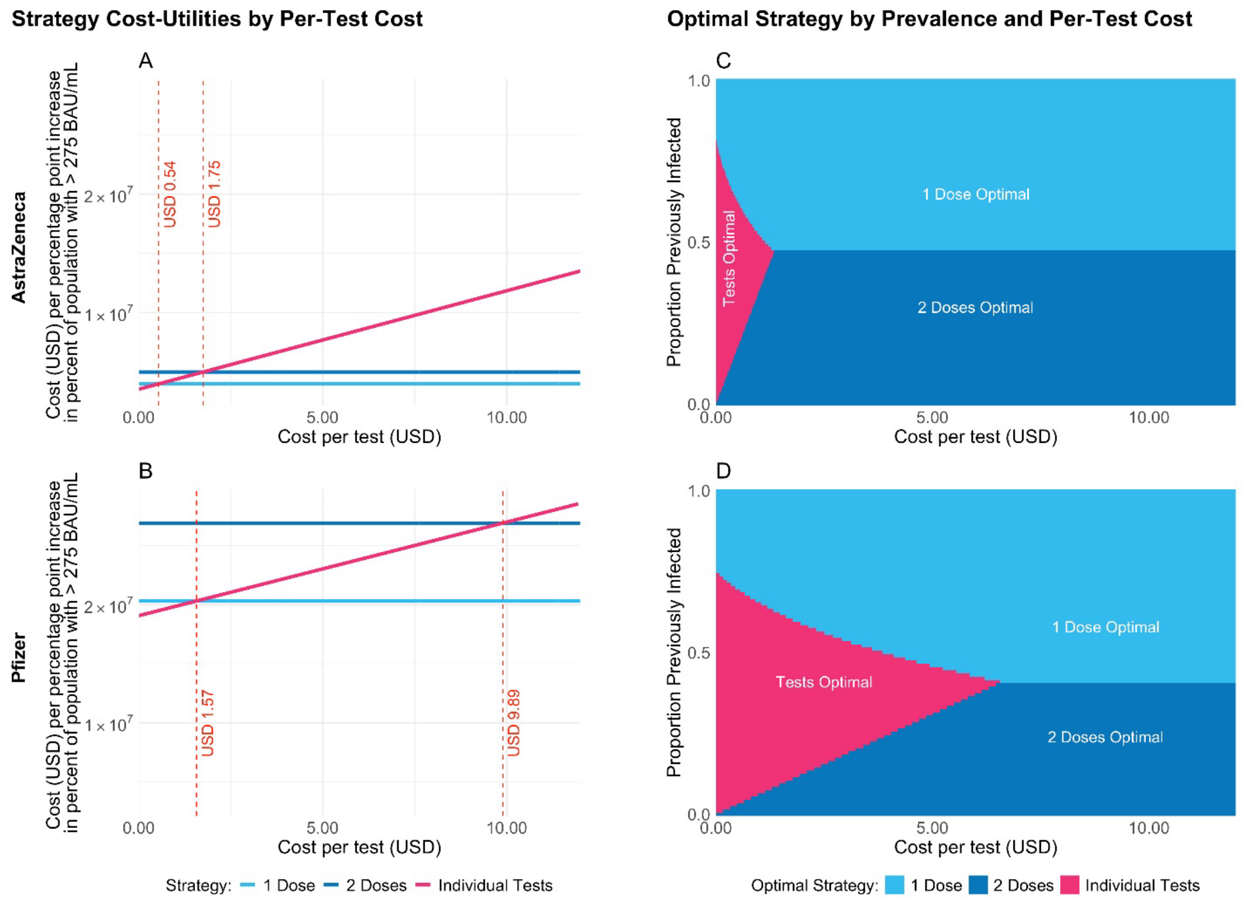

Figure S1. Cost utility analysis results assuming 95% test sensitivity. The cost in USD per unit increase in utility for the three different vaccination strategies for AstraZeneca vs. Pfizer (Panels A and B); the strategy with the lowest cost per unit increase in utility for varying per-test costs and previous infection prevalence for AstraZeneca vs. Pfizer (Panels C and D).

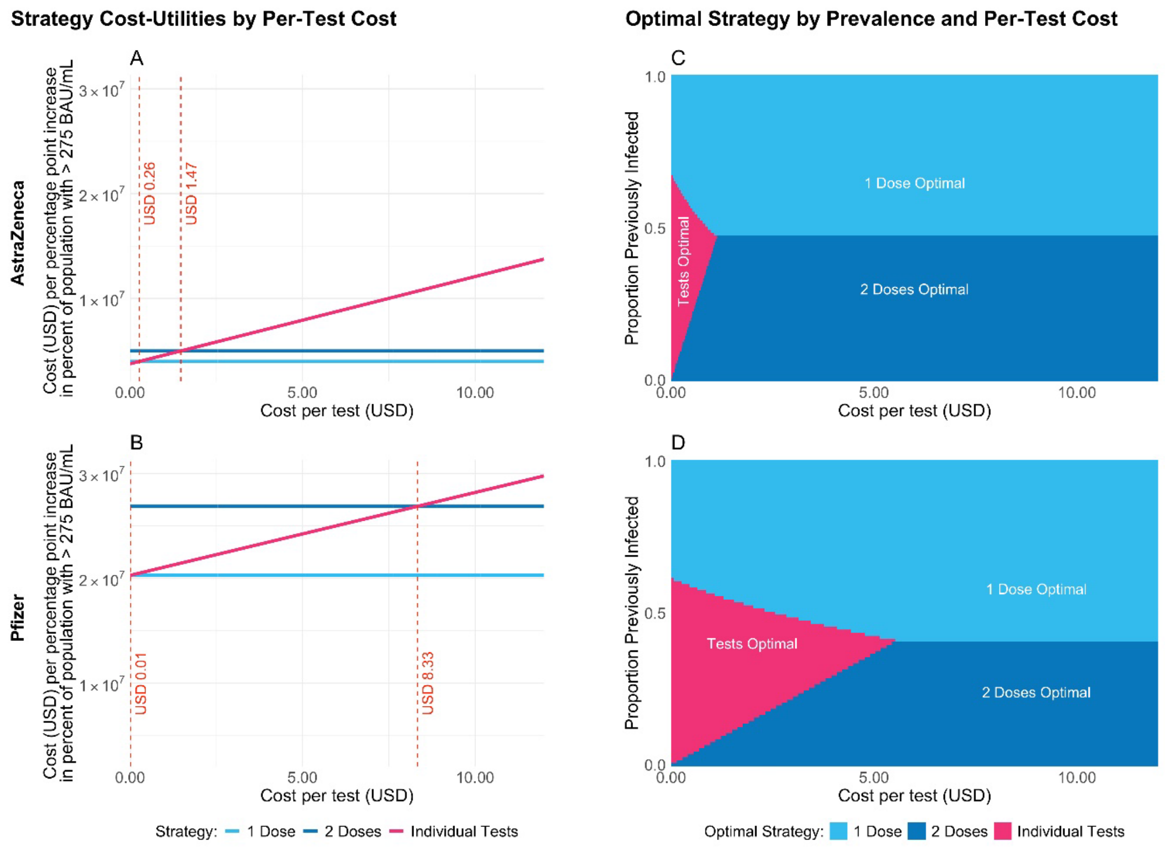

Figure S2. Cost utility analysis results assuming 80% test sensitivity. The cost in USD per unit increase in utility for the three different vaccination strategies for AstraZeneca vs. Pfizer (Panels A and B); the strategy with the lowest cost per unit increase in utility for varying per-test costs and previous infection prevalence for AstraZeneca vs.
Pfizer (Panels C and D).

Parameters and Results for Alternate Utility Function

Instead of the cost per percentage point increase in the immune percentage, we can analyze the cost per log-unit increase in the mean anti-spike level across the population.

Let i now denote the mean log-transformed anti-spike level among the unvaccinated, previously infected population. Similarly, let v now denote the mean log-transformed anti-spike level among the once-vaccinated, not previously infected population and let d denote the mean log-transformed level among the dually exposed population. All other parameter definitions remain the same as before.

From our study data, we now have i = 3.94 log-BAU/mL. We also have that for AstraZeneca, v = 3.86 log-BAU/mL and d = 8.03 log-BAU/mL. Using the reported medians from Wheeler et al. [5], we have v = 4.26 log-BAU/mL and d = 8.37 log-BAU/mL for Pfizer.

Key results for this utility function with Se = 1 are shown in Figure S3. (Note that the x-axis extends to negative numbers, to show that the testing cost at which the Individual Testing Strategy would become optimal for Pfizer is now *less* than 0 USD per test.)

Since comparisons between the 2-Dose and the Individual Testing strategies do not depend on i, v, or d, we obtain the same value of c^*_{test} as before, but $c^\dagger_{\text{test}}$ is now different.

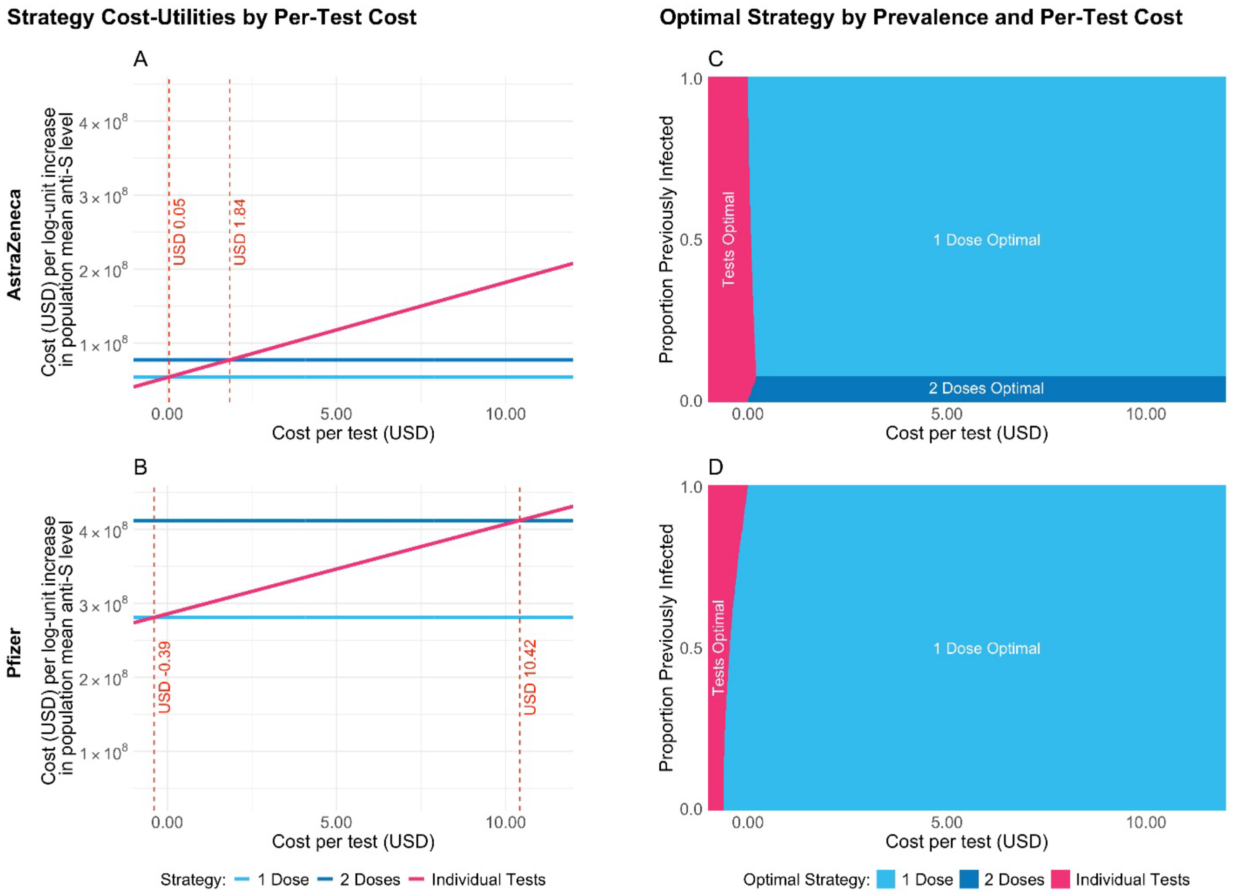

Figure S3. The cost in USD per log-unit increase in population mean antibody level, by strategy for each vaccine (Panels A and B); the strategy with the lowest cost per log-unit increase in utility, by per-test cost and prevalence, for each vaccine (Panels C and D).

References

[1] Simon Merkt et al. "Long-term monitoring of SARS-COV-2 seroprevalence and variants in Ethiopia provides prediction for immunity and cross-immunity". In: *Research Square* (Sept. 2023). doi: 10.21203/rs.3.rs-3307821/v1.

[2] Shuo Feng et al. "Correlates of protection against symptomatic and asymptomatic SARS-COV-2 infection". In: *Nature Medicine* 27.11 (Nov. 2021), pp. 2032–2040. doi: 10.1038/s41591-021-01540-1.

[3] Meghana Sharafudeen et al. *2.2 million COVID-19 vaccines allocated by the COVAX Facility arrive in Ethiopia, marking the start of the country's COVID-19 vaccination campaign*. Mar. 2021. url: https://www.afro.who.int/news/22-million-covid19-vaccines-allocated-covax-facility-arrive-ethiopia-marking-startcountrys.

[4] Aaron O'Neill. *Ethiopia: Age Structure from 2012 to 2022*. Jan. 2024. url: https://www.statista.com/statistics/455134/age-structure-in-ethiopia/.

[5] Sarah E. Wheeler et al. "Differential antibody response to mRNA COVID-19 vaccines in healthy subjects". In: *Microbiology Spectrum* 9.1 (Aug. 2021). doi: 10.1128/spectrum.00341-21.

[6] Anthony McDonnell et al. *Understanding the cost-effectiveness of covid-19 vaccination in Ethiopia*. Apr. 2022. url: https://www.cgdev.org/publication/understandingcost-effectiveness-covid-19-vaccination-ethiopia.

[7] Roche Diagnostics. *Elecsys® Anti-SARS-CoV-2 S*. url: https://diagnostics.roche.com/global/en/products/params/elecsys-anti-sars-cov-2-s.html.
